# Supplementary material for: Prospective, Observational Study of Aflibercept Use in Combination with FOLFIRI in Patients with Metastatic Colorectal Cancer: A Real-World Effectiveness Study
Source: Cancers (Basel). 2024 May 24;16(11):1992. doi: 10.3390/cancers16111992 (PMC11171377; doi:10.3390/cancers16111992)
Supplement: Supplementary file 1 [file cancers-16-01992-s001.zip › cancers-2855876-supplementary.pdf]

Table S1 in Supplementary Materials:

The list of reported serious adverse events (SAE), their degree according to WHO Toxicity Grading Scale and Investigator's assessment of relations to aflibercept.

Nr	Diagnosis	Grade acc. to WHO	Relation to aflibercept
1	Abscess of the area post resection of hepatic metastasis (prothesis inserted to biliary duct)	2	no
2	Asthenia	1	yes
3	Nausea	1	yes
4	Pulmonary embolism	3	no
5	Abdominal pain lower	3	no
6	Neutropenic fever	4	no
7	Hypertension	3	yes
8	Neutropenic fever	3	no
9	Fournier gangrene	4	yes
10	Small intestinal obstruction	4	no
11	Toxic encephalopathy after chemotherapy	3	no
12	Cerebral stroke	5	no
13	Acute kidney injury in course of dehydration due to diarrhoea	4	no
14	Pulmonary embolism	3	no
15	Vascular access site inflammation	3	no
16	Anaemia of malignant disease (the patient was hospitalised for blood red cells transfusion)	3	no
17	Intestinal subobstruction	3	no
18	Acute extrarenal kidney failure	4	yes
